# Supplementary material for: Venomix: a simple bioinformatic pipeline for identifying and characterizing toxin gene candidates from transcriptomic data
Source: PeerJ. 2018 Jul 31;6:e5361. doi: 10.7717/peerj.5361 (PMC6074769; doi:10.7717/peerj.5361)
Supplement: Supplemental Information 6 [file peerj-06-5361-s006.gz › FinalOutput_1E-6/Turripeptide_Ici9/finaltree.pdf]

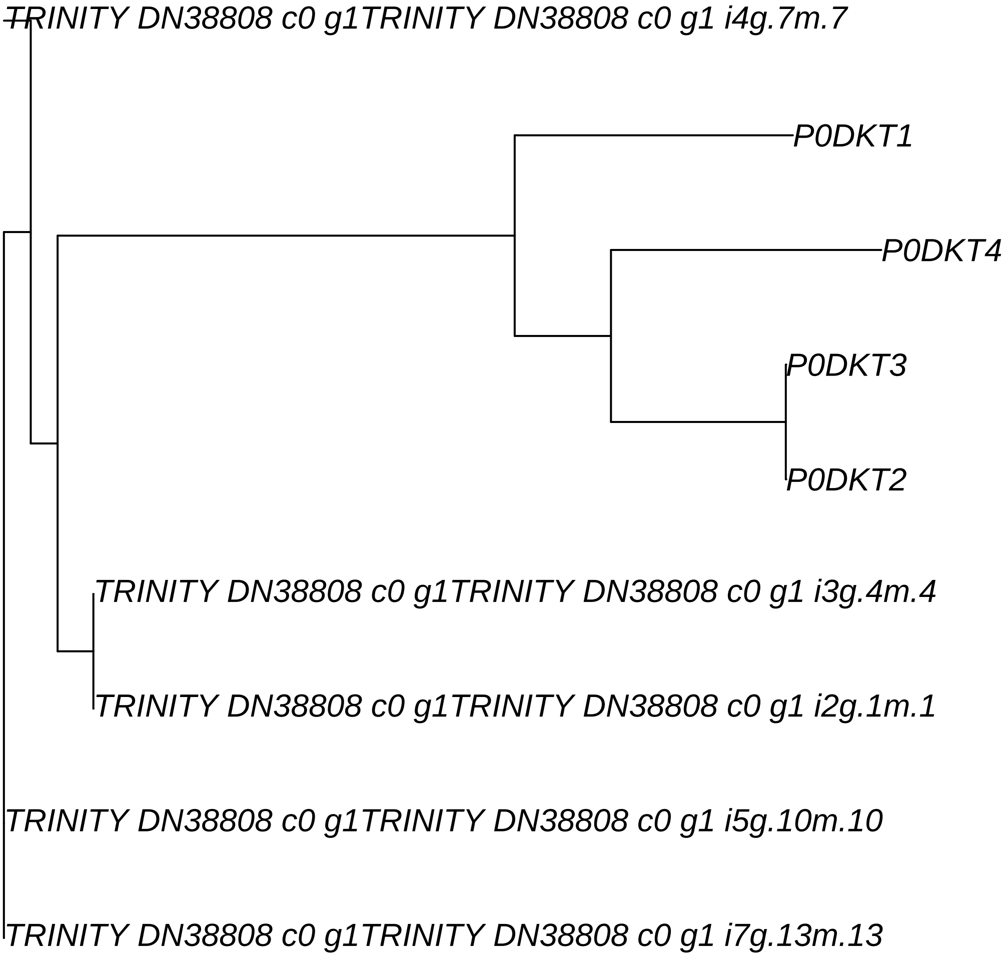
TRINITY DN38808 c0 g1TRINITY DN38808 c0 g1 i4g.7m.7
P0DKT1
P0DKT4
P0DKT3
P0DKT2
TRINITY DN38808 c0 g1TRINITY DN38808 c0 g1 i3g.4m.4
TRINITY DN38808 c0 g1TRINITY DN38808 c0 g1 i2g.1m.1
TRINITY DN38808 c0 g1TRINITY DN38808 c0 g1 i5g.10m.10
TRINITY DN38808 c0 g1TRINITY DN38808 c0 g1 i7g.13m.13
